# Blast-Related Mild Traumatic Brain Injury: Case Report Highlighting the Long-Term Neurocognitive Sequelae Following Blast Exposure in Veterans

**DOI:** 10.7759/cureus.92404

**Published:** 2025-09-15

**Authors:** David Holm, Jennifer Tasarz, Manasi Ponamala, Duc Chung

**Affiliations:** 1 Internal Medicine, St. Bernardine Medical Center, San Bernardino, USA; 2 Biomedical Education, California Health Sciences University College of Osteopathic Medicine, Clovis, USA; 3 Palliative Care, University of California San Francisco, California Health Sciences University, Fresno, USA

**Keywords:** blast injury, chronic dizziness, cognitive effects, post-traumatic brain injury, post-traumatic headache

## Abstract

Traumatic brain injury (TBI) remains a significant public health concern, particularly among military service members exposed to blast-related trauma. While mild TBIs often resolve within weeks, a subset of patients may experience lifelong, persistent, debilitating symptoms. We report a case of a previously healthy male combat medic who sustained a blast-related traumatic brain injury during deployment in Afghanistan, with no loss of consciousness or direct head trauma. Despite the initial mild presentation, he has experienced chronic post-concussive symptoms for over 15 years. This patient presented at a follow-up at the Veterans Affairs Traumatic Brain Injury Clinic with worsened symptomatology. The purpose of this case report is to highlight some of the sequelae of traumatic brain injuries endured by veterans in the military and to underscore the complex and chronic nature of blast-related traumatic brain injuries based on an individualized scale.

## Introduction

Traumatic brain injury (TBI) is defined by the National Institute of Neurological Disorders and Stroke as a form of acquired brain injury in which a sudden trauma causes damage to the brain [1]. According to the Centers for Disease Control and Prevention (CDC), approximately 70,000 people died from TBI in 2021 in the United States [2]. Mild traumatic brain injury (mTBI) has emerged as a signature injury among US military service members, often resulting from blast exposures. mTBI can often present as a complex clinical challenge due to its overlapping comorbidities, heterogeneous symptoms, and subtle neuropathological changes [1]. Traumatic brain injury encompasses a spectrum of severity, ranging from mild to severe, with classification typically based on clinical criteria such as the level of consciousness evaluated by the Glasgow Coma Scale (GCS), duration of loss of consciousness, level and duration of confusion, and neuroimaging findings [3].

Military service members and veterans are at increased risk for TBI due to their exposure to combat-related hazards. According to the US Department of Veterans Affairs, approximately 185,000 veterans receiving care through the United States Department of Veterans Affairs(VA) health system have been diagnosed with TBI, the majority of which are classified as mild [4]. During Operation Enduring Freedom and Operation Iraqi Freedom, blast injuries emerged as a leading cause of mTBI among deployed personnel [3]. These injuries can result from a range of mechanisms, including the shock waves generated by an explosion, impact from flying debris, rapid acceleration or displacement of the body, and exposure to heat or toxic chemicals [5]. Symptoms following a mTBI can include headaches, sleep disturbances, cognitive difficulties, visual and spatial dysfunction, emotional lability, and irritability [3]. While many individuals recover within weeks, a significant subset experiences persistent symptoms that can last for months or even years [6]. Importantly, the clinical presentation of blast-related mTBI may differ from that of nonblast injuries, reflecting distinct pathophysiological mechanisms and recovery trajectories [7].

It should be noted that the differences between non-blast-related mTBI and blast-related mTBI are still being analyzed. Furthermore, it is vital to learn more about the varying presentations between these types of cases in order to tailor rehabilitation strategies and treatment for patients affected by blast-induced neurotrauma. Our patient demonstrates a young veteran who has experienced post-concussive symptoms such as headaches, memory issues, and dizziness for over fifteen years since his deployment, and this case signifies the importance of refining our understanding of blast-related mTBI and its long-term impact. As the burden of chronic symptoms persists and the veteran population continues to experience these struggles, there is a growing imperative to support further functional recoveries. 

## Case presentation

A 35-year-old male with no significant past medical history who was deployed as a combat medic in Afghanistan between 2010 and 2011, who had significant exposure to improvised explosive devices (IEDs), rocket-propelled grenade launchers (RPGs), and mortars exploding near his vehicle, resulting in a significant jolt. The patient did not experience a loss of consciousness and denied any direct head trauma during this incident, reporting only a dazed sensation. At the time of exposure, he presented with altered sensorium, post-traumatic amnesia, increased impulsivity, social withdrawal, as well as progressive headaches, hearing loss, and insomnia alongside memory and attention issues. 

It has now been about 15 years since this event, and he continues to experience these symptoms. The patient mentions that he has headaches that may be up to 7/10 on a pain scale and can occur up to twice a week. He reports that the headaches become more frequent and severe with exertion, which he describes as an aching and consistent sensation that can last up to a few hours and is occasionally relieved by ibuprofen. The patient denies any other medications in regard to pain management for these symptoms. Additionally, he reports experiencing increased sensitivity to light, dizziness, and tinnitus of the right ear since the onset of his blast-related injury. Given the history of his presenting dizziness and tinnitus, he was evaluated for Meniere's disease and was put on a trial of meclizine, which did not provide relief of symptoms. It has also been reported that the patient tends to become disoriented to place and situation, in which he may enter a room and forget why he is there and for what reason he entered. Furthermore, the patient mentions difficulty with focused attention on a daily basis, which has impacted his social life tremendously and has caused him to become more withdrawn due to the feeling of embarrassment when he loses focus during conversations in social settings. He mentions that his difficulty with attention has only worsened since his deployment injury. The ringing sensation in his right ear continues to provide him with discomfort and increased loss of hearing in the ipsilateral ear, which has not improved. 

**Figure 1:**
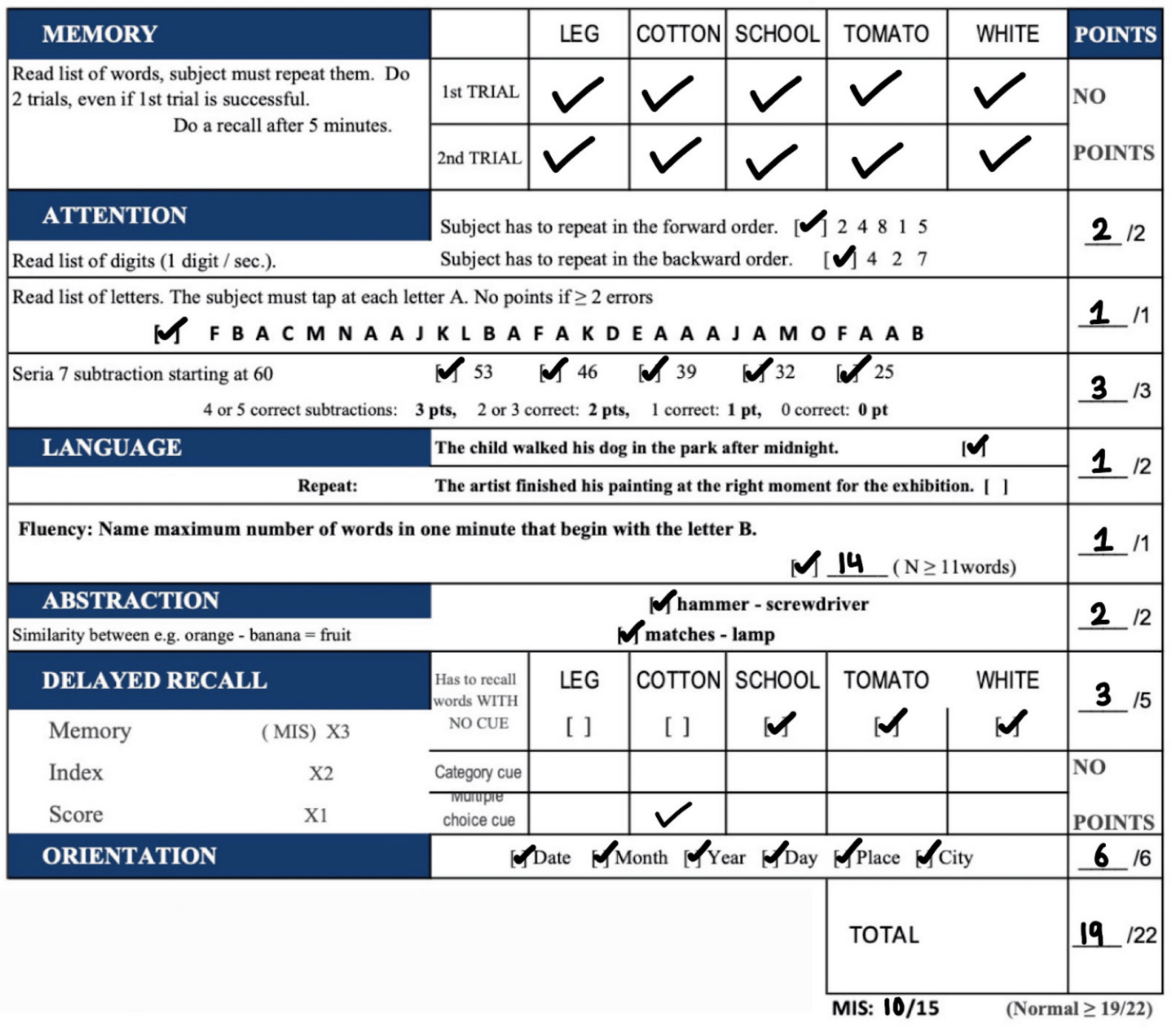
Patient's completed Montreal Cognitive Assessment (MoCA)-BLIND

The patient completed a Montreal Cognitive Assessment (MoCA)-BLIND assessment, as shown in Figure 1, during one of his initial evaluations, where he scored a 19/22, which is considered within normal range. It can be noted that he missed two points while completing the section for delayed recall and one point during the language section.

Furthermore, this patient underwent a neurologic exam during a telehealth visit. The visual exam was limited as this was a televisit. His extraocular movements were intact, and he had no facial asymmetry and no numbness or tingling on his face. There was no weakness or deviation of the tongue on the exam. The patient was able to shrug his shoulders against gravity. On the motor exam, he was able to lift both his arms and his legs against gravity. No dysdiadochokinesia was noted on the exam. His reflexes were not assessed on the exam, given that it was a virtual visit. Overall, his vital signs and results were unremarkable, as was the gross neuro exam on initial visit. No significant lab and imaging were pursued, but he presents with worsening symptoms and subjective complaints of headache and memory issues.

The patient reports a constellation of symptoms consistent with mild traumatic brain injury. However, the evaluation was conducted virtually, which limited the assessment of certain physical functions. The patient is currently attending speech therapy for his memory deficits, as well as occupational therapy. He states that his symptoms have improved since beginning therapy and continues to follow through with the traumatic brain injury clinic.

## Discussion

Traumatic brain injury (TBI) is a major cause of morbidity and mortality among service members and veterans, with blast injury being a common etiology. Symptoms associated with blast-related mild TBI often include post-traumatic headaches, as well as vestibular, auditory, visual, language, and cognitive abnormalities [7]. Table 1 demonstrates some of the common symptoms associated with mild traumatic brain injury, along with the symptoms our patient experienced.

**Table 1:** Common symptoms of mild TBI compared to symptoms reported by the patient TBI - traumatic brain injury

Symptoms of Mild TBI [8]	Reported by the patient	Clinical notes from the patient
Headache	Present	Weekly headaches, worsened with activity
Dizziness	Present	Sensation of the room spinning
Tinnitus	Present	Tinnitus in the right ear
Hearing loss	Present	Diminished hearing in the right ear
Memory deficit	Present	Difficulty remembering words during conversation. Associated with social withdrawal.
Impaired concentration	Present	No additional notes provided
Sleep disturbance	Present	Sleeps approximately three hours per night
Visual dysfunction	Present	Issues with peripheral vision; limited assessment due to virtual visit
More impulsivity	Present	No additional notes provided
Balance dysfunction	Patient denied	Balance was not assessed on the physical exam
Sensitivity to light	Present	Light exacerbates headaches

In this case, the patient has experienced a post-traumatic headache since his blast injury. These headaches occur every week and have not improved over time. He describes them as aching in nature and notes that they become more frequent with physical activity. He takes nonsteroidal anti-inflammatory drugs (NSAIDs) for relief, which provide some benefit. A post-traumatic headache is defined as a headache that begins within seven days of the injury. It is considered acute if it lasts less than three months and chronic if it persists beyond that particular time period [9]. These headaches have been shown to occur more frequently than non-traumatic headaches and may contribute to significant disability in service members and veterans [9]. Although the pathophysiology of post-traumatic headache is still under investigation, proposed mechanisms include cerebrospinal fluid (CSF) changes, oxidative stress, and neuroinflammation [10]. However, these mechanisms may not be exclusive to blast-related mild TBI and could be associated with other forms of injury [10].

It has been noted that both blast-related and non-blast-related mTBI were associated with long-lasting activation of dural mast cells, suggesting a potential role for neuroinflammation in the pathophysiology of post-traumatic headaches [11]. Treatment of post-traumatic headache is typically guided by its clinical presentation. These headaches often present with tension-like or migraine-like features, consistent with this particular case [9]. For tension-type headaches, NSAIDs are recommended for acute episodes, while tricyclic antidepressants (TCAs) may be used for prophylaxis. Migraine-like headaches may also be treated with NSAIDs, and triptans can be added if symptoms persist. For prevention, antihypertensive medications such as propranolol may be effective alongside TCAs [9]. Additional treatment options include transcranial magnetic stimulation and botulinum toxin injections [12]. In refractory cases, peripheral nerve surgery has been shown to result in complete headache resolution for some patients [12].

This patient has also been experiencing cognitive difficulties since his blast-related mTBI. A MoCA-BLIND was administered to assess cognitive function by testing attention, memory, language, recall, abstraction, and orientation of time and place. A normal score is defined as a score of 19 or more, and our patient received a score of 19. However, he demonstrated deficits in two areas. The first deficit was language dysfunction, as he was unable to recall one of the sentences during assessment, which has been associated with frontal lobe pathology [13]. The second was persistent impairment in recall despite cueing, suggesting an encoding deficit involving the Papez circuit, which comprises the hippocampus, fornix, mammillary bodies, thalamus, and cingulate cortex [13]. These findings may indicate specific brain regions affected by blast-related mild traumatic brain injury.

Although the pathophysiology of neurocognitive sequelae in blast injury is not fully understood, it has been found that blast-related mTBI is associated with decreased brain volumes in gray matter regions such as the pallidum and in white matter structures like the corona radiata, which are not typically observed in nonblast TBI [14]. This may explain the higher incidence of neurocognitive symptoms in veterans and service members [14]. In a preclinical study using a mouse model of blast injury, damage was primarily localized to the parietal cortex and hippocampus. Similar findings have been reported in humans, where functional magnetic resonance imaging (fMRI) studies revealed parietal cortex damage in individuals with mechanical or blast-related TBI [15]. These changes may contribute to the neurocognitive symptoms observed in blast-exposed individuals. Another study on human subjects showed that blast-related TBIs were associated with reduced cortical thickness, particularly in those with multiple injuries. This reduction was linked with poorer executive functioning, depending on severity [16]. However, research on blast-related mTBI remains fairly inconsistent, and not all cases lead to cognitive decline [17]. To address cognitive dysfunction in affected veterans and service members, cognitive rehabilitation is recommended, especially when delivered by a multidisciplinary team and initiated early after injury [6, 17]. 

Dizziness and balance dysfunction are also common features of blast-related mTBI [18]. Several mechanisms may lead to dizziness following mTBI. One such mechanism involves white matter and axonal injury. In a review of four patients exposed to blast injuries, all exhibited some degree of microhemorrhages or vascular anomalies on neuroimaging [19]. Three of the four patients had associated vestibular dysfunction, and all four had balance dysfunction, suggesting possible central nervous system (CNS) involvement [19]. Peripheral vestibular dysfunction is another possible mechanism that contributes to mTBI. In one study, rodents exposed to blast levels comparable to those of IEDs showed significant damage to vestibular structures, including loss of stereocilia [20]. Additionally, patients with chronic dizziness following blast exposure demonstrate vestibular testing abnormalities associated with peripheral etiologies [21]. Another potential cause of dizziness is disruption of otoconia (ear stones), which can lead to benign paroxysmal positional vertigo (BPPV) [22]. 

Our patient described his dizziness as vertigo-like and accompanied by significant nausea. A full vestibular and balance assessment was not completed due to the limitations of a telehealth format of the visit. This limited our ability to further assess dizziness. Treatment options for patients with mTBI-related dizziness can vary. The United States Department of Veterans Affairs recommends individualized vestibular rehabilitation based on exam findings such as increased motion sensitivity, impaired gaze stabilization, functional gait abnormalities, or cervicogenic dysfunction [8]. Patients diagnosed with BPPV may benefit from canalith repositioning therapy [22]. Another emerging treatment approach involves the use of nociceptin/orphanin FQ peptide (NOP) receptor antagonists. This neuropeptide is upregulated following TBI and expressed in vestibular nuclei. In rodent models, NOP receptor antagonists improved vestibular motor performance after blast exposure [23]

Due to the often debilitating consequences of blast-related mild TBI, advanced neuroimaging techniques play a critical role in revealing underlying brain changes. One study found that veterans with blast-related mTBI had reduced brain volumes in regions such as the superior corona radiata, globus pallidus, substantia nigra, nucleus accumbens, internal capsule, and cerebellar peduncle. Such brain volume changes were associated with decreases in cognitive function [14]. Another report showed cortical thinning on magnetic resonance imaging (MRI) involving the anterior frontal lobes in military service members with blast-related mTBI. Damage to such areas has been associated with executive function and decision making [24].

The patient was evaluated through telehealth, which limited the ability to perform a comprehensive neurological examination by a medical provider. Key components such as reflexes, muscle strength, and sensory testing could not be directly assessed. During the virtual visit, the patient reported diminished peripheral vision. This concern was challenging to evaluate thoroughly in a remote setting. As a result, potential neurologic deficits may have gone undetected.

Additionally, a complete vestibular and balance assessment was not performed, making it difficult to determine whether the patient's dizziness stemmed from a peripheral or central origin. For concerns involving neurological function, an in-person evaluation is strongly recommended to allow for a full and accurate assessment.

## Conclusions

The current case underscores the long-term impact of blast-related traumatic brain injuries on military veterans and the need for comprehensive, multidisciplinary evaluation and treatment due to their subsequent neurotrauma symptoms. Also, this case shows that despite a normal MoCA-BLIND, patients with blast-related mTBI may still report cognitive issues that negatively impact their lives. Symptoms of blast-related mTBI typically dissipate after several weeks, but our patient case highlights the chronicity of disabling symptoms that may persist for many years after the initial insult. It is essential for clinicians to recognize these symptoms, especially in this particular patient population, so that appropriate rehabilitation strategies, individualized treatment, and further research can be initiated into the distinct experiences correlated with blast-induced mild traumatic brain injuries. This includes specific rehabilitation for memory-related symptoms and dizziness alongside medication management.
